# Chemical Composition and the Anticancer, Antimicrobial, and Antioxidant Properties of *Acacia* Honey from the Hail Region: The in vitro and in silico Investigation

**DOI:** 10.1155/2022/1518511

**Published:** 2022-08-04

**Authors:** Walid Sabri Hamadou, Nouha Bouali, Riadh Badraoui, Ramzi Hadj Lajimi, Assia Hamdi, Mousa Alreshidi, Mitesh Patel, Mohd Adnan, Arif Jamal Siddiqui, Emira Noumi, Visweswara Rao Pasupuleti, Mejdi Snoussi

**Affiliations:** ^1^Department of Biology, College of Science, University of Hail, P.O. Box 2440, Ha'il, Saudi Arabia; ^2^Section of Histology Cytology, Medicine Faculty of Tunis, University of Tunis El Manar, La Rabta 1007, Road Djebal Lakhdhar, Tunis 1007, Tunisia; ^3^Department of Histo-Embryology and Cytogenetics, Medicine Faculty of Sfax, University of Sfax, Road of Majida Boulia, Sfax 3029, Tunisia; ^4^Department of Chemistry, College of Science, University of Ha'il, P.O. Box 2440, Ha'il 81441, Saudi Arabia; ^5^Laboratory of Water, Membranes and Environmental Biotechnologies, Center of Researches and Water Technologies, P.O. Box 273, Soliman 8020, Tunisia; ^6^Laboratoire de Développement Chimique Galénique et Pharmacologique des Médicaments, Faculté de Pharmacie, Monastir 5000, Tunisia; ^7^Molecular Diagnostic and Personalized Therapeutics Unit, University of Ha'il, Ha'il 2440, Saudi Arabia; ^8^Department of Biotechnology, Parul Institute of Applied Sciences and Centre of Research for Development, Parul University, Vadodara 391760, Gujarat, India; ^9^Laboratory of Bioresources: Integrative Biology and Recovery, High Institute of Biotechnology University of Monastir, Monastir 5000, Tunisia; ^10^Department of Biomedical Sciences and Therapeutics, Faculty of Medicine & Health Sciences, Universiti Malaysia Sabah, Kota Kinabalu, Sabah 44800, Malaysia; ^11^Centre for International Collaboration and Research, Reva University, Rukmini Knowledge Park, Kattigenahalli, Yelahanka, Bangalore, Karnataka 560064, India; ^12^Laboratory of Genetics, Biodiversity and Valorisation of Bioresources, High Institute of Biotechnology University of Monastir, Monastir 5000, Tunisia

## Abstract

In consideration of the emergence of novel drug-resistant microbial strains and the increase in the incidences of various cancers throughout the world, honey could be utilized as a great alternative source of potent bioactive compounds. In this context, this study pioneers in reporting the phytochemical profiling and the antimicrobial, antioxidant, and anticancer properties of *Acacia* honey (AH) from the Hail region of Saudi Arabia, assessed using *in vitro* and molecular docking approaches. The phytochemical profiling based on high-resolution liquid chromatography-mass spectrometry (HR-LCMS) revealed eight compounds and three small peptide-like proteins as the constituents. The honey samples exhibited promising antioxidant activities (DPPH-IC_50_ = 0.670 mg/mL; ABTS-IC_50_ = 1.056 mg/mL; *β*-carotene-IC_50_ > 5 mg/mL). In the well-diffusion assay, a high mean growth inhibition zone (mGIZ) was observed against *Staphylococcus aureus* (48.33 ± 1.53 mm), *Escherichia coli* ATCC 10536 (38.33 ± 1.53 mm), and *Staphylococcus epidermidis* ATCC 12228 (39.33 ± 1.15 mm). The microdilution assay revealed that low concentrations of AH could inhibit the growth of almost all the evaluated bacterial and fungal strains, with the minimal bactericidal concentration values (MBCs) ranging from 75 mg/mL to 300 mg/mL. On the contrary, high AH concentrations were required to kill the tested microorganisms, with the minimal bactericidal concentration values (MBCs) ranging from approximately 300 mg/mL to over 600 mg/mL and the minimal fungicidal concentration values (MFCs) of approximately 600 mg/mL. The AH exhibited effective anticancer activity in a dose-dependent manner against breast (MCF-7), colon (HCT-116), and lung (A549) cancer cell lines, with the corresponding IC_50_ values of 5.053 *μ*g/mL, 5.382 *μ*g/mL, and 6.728 *μ*g/mL, respectively. The *in silico* investigation revealed that the observed antimicrobial, antioxidant, and anticancer activities of the constituent compounds of AH are thermodynamically feasible, particularly those of the tripeptides (Asp-Trp-His and Trp-Arg-Ala) and aminocyclitol glycoside. The overall results highlighted the potential of AH as a source of bioactive compounds with significant antimicrobial, antioxidant, and anticancer activities, which could imply further pharmacological applications of AH.

## 1. Introduction

The emergence of novel drug-resistant microbial strains and the high prevalence of various cancers have heavily burdened the existing healthcare system. The main challenge is to discover novel therapeutic strategies. Complementary medicine using natural products or their derivative phytochemicals could serve as a great alternative for overcoming these healthcare-related concerns, and honey could be an interesting choice in this regard [1]. Honey and its products have been valorized for their high nutritional value and medicinal properties for a long time. Owing to its therapeutic value, honey is used as an essential ingredient in several formulations of folk medicine. The diversity of the specific phytochemical compositions of honey could provide valuable bioactive molecules to be used in the treatment of various cancers and infections with drug-resistant bacterial strains. Certain highly antioxidant and bioactive constituent molecules in honey have been reported to exhibit prominent therapeutic results against several forms of cancers. So far, over 200 constituent compounds have been reported in honey. This complexity of honey could explain its antimicrobial and anticancer activities and its capacity to modulate oxidative stress. The most common bioactive compounds reported in honey, which are considered responsible for its anticancer activity, are flavonoids and phenolic acids [2]. These compounds exhibit several mechanisms for anticancer activity, including apoptosis, inhibition of the tumor necrosis factor, and antiproliferative, immunomodulatory, and anti-inflammatory effects [2]. Honey samples derived from several regions have been investigated for their antioxidant, antimicrobial, and anticancer activities, with different results reported for different subtypes and various floral and geographical origins of honey [3]. The various kinds of honey derived from Saudi Arabia have been particularly highlighted for their interesting displays of biological activity [4–7]. In the Kingdom of Saudi Arabia, beekeeping constitutes an important economic activity, with several varieties of honey produced as this region has abundant natural flora most suitable for beekeeping. Talh or *Acacia* is the dominant bee plant in this region. This plant belongs to the subfamily Mimosoideae under the family Fabaceae, which comprises several species, including *Acacia albida, Acacia asak, Acacia ehrenbergiana, Acacia etbaica, Acacia johnwoodii, Acacia oerfota,* and *Acacia tortilis* [8].

*Acacia* honey (AH) is quite popular in Saudi Arabia owing to its nutritional value and medicinal properties. The composition and properties of AH mainly depend on the geographical and floral origin of the honey and also on the environmental factors, including seasons. Several studies have highlighted that the chemical composition of honey may even vary with climatic conditions and soil composition [9]. Recent studies revealed that honey samples obtained from different altitudes in the same region exhibited different phytochemical compositions, particularly the levels of total phenols and flavonoids, which could lead to different biological activities. The antioxidant, antimicrobial, and anticancer potentials of AH have been revealed in several studies. The reported results varied according to the bioactive phytochemical composition of AH, which mainly depends on the geographical origin of the honey sample [7]. Therefore, this study aimed at investigating, for the first time, the phytochemical composition of AH from the Hail region using the HR-LCMS technique to evaluate the antioxidant potential of the selected honey and estimate its anticancer activity against human lung, breast, and colon cancer cell lines. In addition, the antimicrobial potential of the selected AH against several bacterial strains, yeast, and molds was investigated using the well-diffusion and microdilution assays. The targeted biological activities were also assessed by studying the molecular interactions with the selected receptors using the molecular docking approach.

## 2. Materials and Methods

### 2.1. Honey Sampling

Talh (*Acacia* sp.) honey samples were collected directly from the beekeeper in the melliferous areas of the Hail region. The collected honey samples were stored at 4°C in glass jars in the dark until to be used for subsequent analyses.

### 2.2. Phytochemical Profiling of *Acacia* Honey

#### 2.2.1. Identification of Bioactive Compounds Using the HR-LCMS Technique

In order to determine the phytochemistry of AH, chromatographic analysis was performed using the UHPLC-PDA-detector mass spectrophotometer (HR-LCMS 1290 INFINITY UHPLC System, Agilent Technologies, Santa Clara (CA, USA), as described previously by Adnan et al. [10]. A 10-*μ*L aliquot from the honey sample was injected into the SBC18 column (2.1 mm × 50 mm; particle size 1.8 *μ*m). The elution was performed using 1% formic acid in deionized water (solvent A) and acetonitrile (solvent B) at a flow rate of 0.350 mL/min. The MS detection was performed using MS Q-TOF in positive and negative atmospheric pressure chemical ionization modes. PubChem was employed as the main tool for the identification of the phytochemical constituents in the honey samples.

#### 2.2.2. Determination of Total Phenols, Flavonoids, and Tannins

Total phenols, flavonoids, and tannins were quantified using the standard protocols described in previous reports [11, 12].

### 2.3. Antioxidant Activities

The antioxidant activities were evaluated using three tests DPPH, ABTS, and *β*-carotene bleaching tests. The test protocols used were obtained from previous reports [13, 14].

#### 2.3.1. DPPH Radical-Scavenging Activity

The DPPH (2,2-diphenyl-1-picryl-hydrazyl-hydrate) (Sigma-Aldrich Milano, Italy) free radical-scavenging activities of the honey samples were determined using an antioxidant assay based on electron-transfer reaction. In this assay, DPPH is reduced in the presence of an antioxidant molecule. Several dilutions of the honey were incubated with DPPH for 30 min at room temperature. Ascorbic acid was used as the standard control. The variation in the color of DPPH was assessed based on spectrophotometric analysis at 515 nm. The antioxidant activity was expressed as IC_50_ (mg/mL) and calculated using the following formula:

(1) $$\begin{array}{c} \text{DPPH}-\text{scavenging activity}\left(\%\right)=\frac{\left(A_{0}-A_{1}\right)}{A_{0}}\times 100, \end{array}$$

where *A*_0_ denotes the absorbance of the control and *A*_1_ denotes the absorbance of the sample.

#### 2.3.2. ABTS Radical-Scavenging Activity

The ABTS (2,2-azino-bis (3-ethylbenzothiazoline-6-sulfonic acid); Sigma-Aldrich Milano, Italy) radical-scavenging activity of the honey samples was assessed. In order to generate ABTS^+^, 7 mM ABTS was allowed to react with 2.45 mM K_2_S_2_O_8_ for 12 h in the dark at room temperature. Several concentrations of the honey were mixed with 900 *μ*L of the solution-containing ABTS^+^, and the mixture was incubated for 30 min. The antioxidant activity was expressed as IC_50_ (mg/mL), representing the AH concertation scavenging 50% of ABTS^+^. The ABTS-scavenging activity was calculated using the following formula:

(2) $$\begin{array}{c} \text{ABTS}-\text{scavenging activity}\left(\%\right)=\frac{\left(A_{0}-A_{1}\right)}{A_{0}}\times 100, \end{array}$$

where *A*_0_ denotes the absorbance of the control and *A*_1_ denotes the absorbance of the sample.

#### 2.3.3. *β*-Carotene/Linoleic Acid Method

The *β*-carotene method was performed as described by Ikram et al. [15], and the inhibition of the volatile organic compounds and the conjugated diene hydroperoxides arising from linoleic acid oxidation was measured. In the reaction, the free radical linoleic acid attacked the highly unsaturated *β*-carotene in the presence of antioxidants (*Acacia* honey in this case). The prepared emulsion mixture was incubated at 50°C in the dark, followed by measuring the absorbance at 470 nm immediately (*t* = 0 min) and after 2 h of incubation (*t* = 120 min). Ascorbic acid was used as the standard for comparison. The antioxidant activity of honey was estimated using the following equation:

(3) $$\begin{array}{c} \text{PI}\left(\%\right)=\left(\frac{A-\beta -\text{carotene }T_{120}}{A-\beta -\text{carotene }t_{0}}\right)\times 100. \end{array}$$

### 2.4. Antimicrobial Activity of the Obtained Crude Extract

#### 2.4.1. Well-Diffusion Assay

One mL of the honey sample to be evaluated (1 mL = 1.0653 g) was diluted in 5% dimethyl sulfoxide (DMSO) to prepare solutions with the final concentration ranging from 532.65 mg/mL to 26 mg/mL. Subsequently, the well-diffusion assay was performed to determine the diameter of the growth inhibition zone on agar medium (Mueller–Hinton for bacteria, Sabouraud Chloramphenicol agar for yeasts, and potato dextrose agar for molds) using the protocols described by Noumi et al. [12, 16]. The evaluated strains were obtained from a huge microbial collection available in the microbiology laboratory of our college and included the following twelve bacteria: *Pseudomonas aeruginosa* (clinical strain, SP-40), *Staphylococcus aureus* ATCC 29213, *S. epidermidis* ATCC 12228, *Escherichia coli* ATCC 10536, *Klebsiella pneumoniae* (clinical strain, 140), *E. coli* (clinical strain, 217), *S. aureus* (clinical strain), *S. sciuri* (environmental strain), *Serratia marcescens*, *Acinetobacter baumannii* (clinical strain, 146), methicillin-resistant *S. aureus* (MRSA clinical strain, 136), and *Enterobacter cloacae* (clinical strain 155). The antifungal activity of the AH to be evaluated was investigated against the following five yeasts and two molds: *Candida albicans* ATCC 20402, *Saccharomyces cerevisiae* (instant yeast), *C. vaginalis* (clinical strain, 136), *C. guilliermondii* ATCC 6260, *C. tropicalis* ATCC 1362, *Aspergillus fumigatus* ATCC 204305, and *A. Niger*. The antibacterial activity was evaluated using the agar well-diffusion assay. Using a sterile borer (diameter 7 mm), four wells were created in the agar plate and filled with 100 *μ*L of AH at different concentrations (100%, 75%, 50%, and 25%). The pure colonies of microorganisms that appeared on the appropriate agar media were selected to prepare homogenous (bacterial/fungal) suspensions. A cotton swab was used for inoculating fresh Petri dishes.

The treated Petri dishes were maintained at 4°C for 1 h and then incubated at 37°C for the next 24 h. The antimicrobial activity was evaluated by measuring the diameter of the growth inhibition zone around the wells. All tests were performed in triplicate, and the mean diameter of the inhibition zone was used as the final diameter value. The results obtained were interpreted using the scheme proposed by Parveen et al., which was as follows: no activity growth inhibition zone 0 (GIZ 0), low activity (GIZ: 1–6 mm), moderate activity (GIZ: 7–10 mm), high activity (GIZ: 11–15 mm), and extremely high activity (GIZ: 16–20 mm). Ampicillin (10 mg/mL; 10 *μ*L/disc) and amphotericin B (10 mg/mL; 10 *μ*L/disc) treatments were performed in the control experiments.

#### 2.4.2. Microdilution Assay

The microdilution method was adopted to determine the MICs and MBCs (minimum bactericidal concentrations)/MFC values of the honey samples, as described previously [17]. First, a stock solution was prepared in 5% DMSO. Next, twofold serial dilutions of the honey samples were prepared in the wells of 96-well plates, beginning from 532.65 mg/mL to 26 mg/mL, using Mueller–Hinton broth for bacteria and Sabouraud Chloramphenicol broth for yeasts and molds. The microbial inoculum (5 *μ*L) was then added to each well of the microtiter plate containing 0.1 mL of the serially diluted honey. This was followed by incubation at 37°C for 24 h. The minimum inhibitory concentration (MIC) was defined as the lowest concentration of the compound that could inhibit microbial growth. In order to determine the MBC/MFC values, 3 *μ*L of the medium was removed from the wells with no visible growth and inoculated on Mueller–Hinton/Sabouraud Chloramphenicol agar plates. After 24 h of incubation at 37°C, microbial growth was observed. The concentration at which the microorganisms were killed (no growth) was recorded as the minimum bactericidal/fungicidal concentration. The MBC/MIC and MFC/MIC ratios were used to determine the activity of the honey samples as described in previous reports [18, 19].

### 2.5. Anticancer Assay Using MTT Assay

The anticancer potential of AH was evaluated against three human cancer cell lines using the MTT (3-(4,5-dimethylthiazolyl-2)-2,5 diphenyl tetrazolium bromide) assay. The three cell lines used in this study, namely, the lung cancer (A549), breast cancer (MCF-7), and colon cancer (HCT-116) cell lines, were provided by the National Centre for Cell Science (NCCS), Pune, India. Doxorubicin (Sigma, India) was used as the reference drug in the MTT assay. The cells were maintained at 37°C, 5% CO_2_, and 80% humidity in 25-cm^2^ flasks containing Dulbecco's modified Eagle medium (DMEM) supplemented with 10% fetal bovine serum (FBS), 10,000 U/mL penicillin, and 5 mg/mL streptomycin (Hi-Media, India). When 80% confluence was reached, the cells were seeded in the wells of a 96-well plate at a density of 1 × 10^5^ cells per well, followed by incubation at the conditions stated above. The cells were stained with approximately 0.4% trypan blue stain (Hi-Media, India) to estimate the number of viable cells using a hemocytometer. All treatments were performed in triplicate, each for 24 h and with different concentrations of AH (2%, 4%, 6%, 8%, and 10%) prepared by diluting AH in complete media and sterilized using filtration (0.22 *μ*m filter). After incubation, the plate was removed from the incubator, and the medium containing AH was aspirated and then washed with phosphate-buffered saline (PBS). Afterward, the cells were incubated with 100 *μ*L of the MTT (Hi-Media, India) solution (5 mg/mL) for 4 h. Subsequently, 100 *μ*L of DMSO was added for crystal solubilization, and the absorbance was recorded at the wavelengths of 570 nm and 630 nm using an ELISA reader. The percentage growth inhibition was calculated after subtracting the background and the blank, and the concentration of the evaluated drug required to inhibit cell growth by 50% (IC_50_) was calculated from the dose-response curve for the respective cell line as described in a previous report [20].

### 2.6. The *In Silico* Analysis

The targeted biological activities were confirmed using the *in silico* molecular docking and interaction approach. The macromolecules identified in the composition of honey samples were obtained from RCSB. The macromolecules with the PDB IDs 1JIJ, 2XCT, 2QZW, and 1HD2 were subjected to the assessment of antibacterial, antifungal, and antioxidant activities. Similarly, 4UYA, 1JNX, and 4BBG were evaluated for their potential anticancer effect on colon, breast, and lung cancers, respectively. The chemical structures of all macromolecules were drawn using ChemDraw and saved in the. sdf format. The docking approach was based on the CHARMm force field after processing the receptors by adding polar hydrogens and Kollman charges and removing the crystal water molecules [21, 22] in AutoDock Vina and DS visualizer. The binding affinity and hydrogen bond assessment calculations were performed as described previously [21–24].

### 2.7. Statistical Analysis

All experiments and measurements were conducted in triplicate, and the results were presented as mean values ± SD (standard deviations). ANOVA, Duncan's, and Bonferroni tests were performed using SPSS 16.0 and GraphPad Prism 5.0. The means of the result values obtained in the tests were also evaluated with the least significant differences test at *p* < 0.05.

## 3. Results

### 3.1. Phytochemical Profiling of *Acacia* Honey

High-resolution liquid chromatography-mass spectrometry (HR-LCMS) was performed to determine the chemical composition of AH. This technique enabled the separation and identification of the phytoconstituents based on their retention time, database difference (library), experimental m/z, MS/MS fragments, metabolite class, and the proposed compounds. The MS data were generated in both negative and positive ionization modes. The complete list is provided in Table 1. The technique identified small peptide-like proteins in AH (one dipeptide and two tripeptides), with the respective molecular weights ranging from 271.1674 g/mol to 456.1732 g/mol.

**Figure 1:**
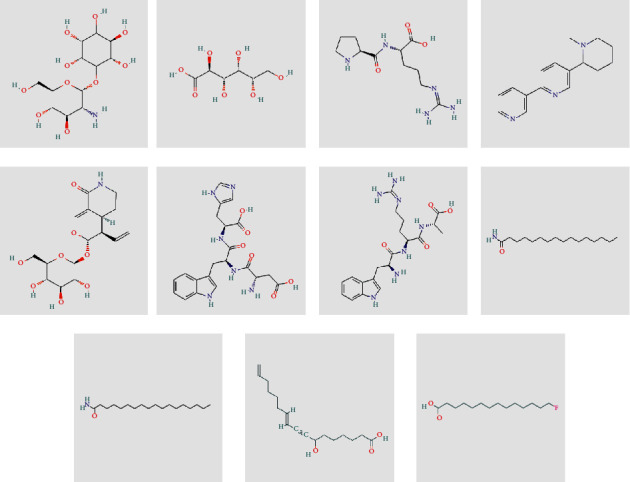
Chemical structure of eleven phytochemical compounds and small peptides identified in *Acacia* honey by using HR-LCMS technique.

The negative and positive runs identified 9 compounds belonging to different chemical classes, including 6-(alpha-D-glucosaminyl)-1D-myo-inositol, L-gulonate, anabasamine, bakankoside, palmitic amide, stearamide, acetylenic acids, 10,16-heptadecadien-8-ynoic acid, 7-hydroxy, (E), and 14-fluoro-myristic acid. The chemical structures of the identified molecules are depicted in Figure 1.

### 3.2. Antimicrobial Activities of *Acacia* Honey

**Figure 2:**
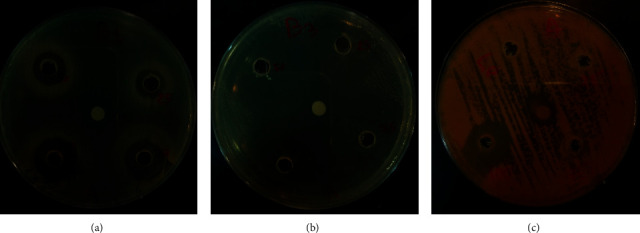
Antimicrobial activity of *Acacia* honey. (a) *P. aeruginosa* (clinical strain, SP-40), (b) *S. epidermidis* ATCC 12228 as compared to ampicillin (10 mg/ml), and (c) *S. cerevisiae* as compared to amphotericin B (10 mg/ml).

The mean diameters of the growth inhibition zones observed for the evaluated AH samples against 12 bacteria, 5 yeasts, and 2 molds are listed in Table 2. The highest mGIZ value for all the evaluated bacteria was obtained when honey was used at 100% concentration. At this concentration, *S. aureus* (clinical strain), *E. coli* (clinical strain 217), and *S. epidermidis* ATCC 12228 were revealed as the most sensitive strains, with the corresponding mGIZ values of approximately 40.67 ± 0.57 mm, 50.33 ± 0.57 mm, and 40.33 ± 0.57 mm, respectively (Figure 2).

The most resistant bacterial strains were *A. baumannii* (clinical strain, 146), which exhibited an mGIZ value of approximately 6.00 ± 0 mm. The evaluated AH could not better inhibit the growth of any of the selected yeasts and molds compared to the reference antifungal drug (amphotericin B), except for the growth of the *S. cerevisiae* strain, for which the mGIZ values ranged from 15.00 ± 0 mm at 75% of AH to 20.00 ± 0 mm when pure AH (100%) was used (Figure 2).

The findings of the microdilution method revealed that the lowest MIC values of 75 mg/mL were obtained against the staphylococcal species (*S. aureus*, *S. epidermidis*, and *S. scuri*) and *S*. *marscescens*. On the other hand, 300 mg/mL of AH was required to inhibit the growth of methicillin-resistant *S. aureus* strain. The MIC values of the fungal strains ranged from 150 mg/mL to 300 mg/mL. Higher concentrations of AH were required to completely kill the bacteria in all evaluated strains (the MBC values ranged from 300 mg/mL to 600 mg/mL). In addition, AH concentrations higher than 600 mg/mL were required to reproduce a fungicidal action against Candida and *Saccharomyces* strains. The results obtained using the scheme proposed by Gatsing et al. [19] revealed that AH exhibited bactericidal activity against most Gram-positive and Gram-negative bacteria, with the corresponding MBC/MIC ratios of ≤4. Similarly, the evaluated honey samples exhibited fungistatic activity against the selected yeast, with an MFC/MIC ratio of ≤4. All the above data are provided in Table 3.

### 3.3. Antioxidant Potential of *Acacia* Honey

The total phenolics, flavonoid, and tannin contents in the AH samples are listed in Table 4. Among the three different approaches used to assess the antioxidant activity of AH, the highest scavenging activity was observed using the DPPH assay, which revealed an IC_50_ value of 0.670 mg/mL, followed by the ABTS and *β*-carotene assays, which revealed the IC_50_ values of 1.065 mg/mL and >5 mg/mL, respectively.

### 3.4. Anticancer Activity of *Acacia* Honey

**Figure 3:**
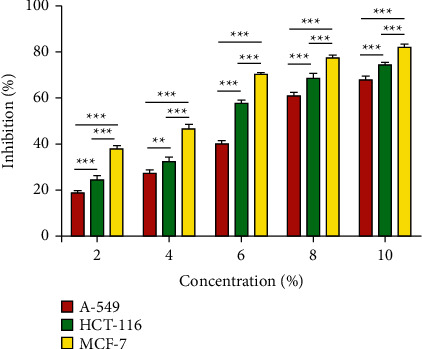
Effect of the *Acacia* honey cytotoxicity on breast (MCF-7), lung (A549), and colon (HCT-116) cancer cell lines according to concentration variation. Error bars indicate SEM (standard error of the mean) of three independent experiments. Significance; ns > 0.05, ^*∗*^*p* < 0.05, ^*∗∗*^*p* < 0.005, ^*∗∗∗*^*p* < 0.0005.

The results of the cytotoxicity evaluation of AH against breast cancer (MCF-7), lung cancer (A549), and colon cancer (HCT-116) cell lines are summarized in Figure 3. AH exhibited promising anticancer activity against the selected cancer cell lines, with the corresponding IC_50_ values of 5.053 *μ*g/mL, 5.382 *μ*g/mL, and 6.728 *μ*g/mL against the breast, colon, and lung cancer cell lines.

### 3.5. The *in silico* Analysis

**Figure 4:**
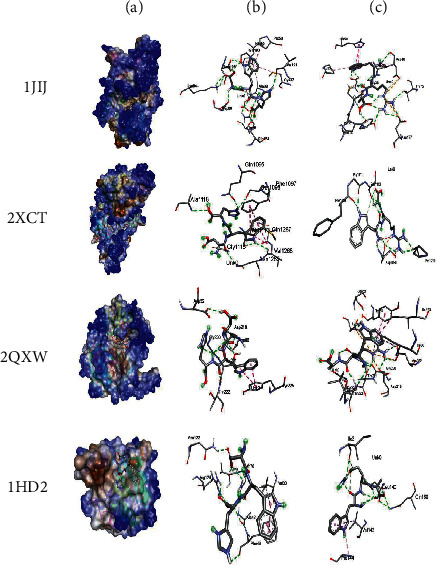
3D illustrations of the selected honey compounds (5 and 6), which possessed the highest binding scores and the targeted receptors (a); the corresponding closest 3D interactions for compounds nos. 5 (b) and 6 (c).

**Figure 5:**
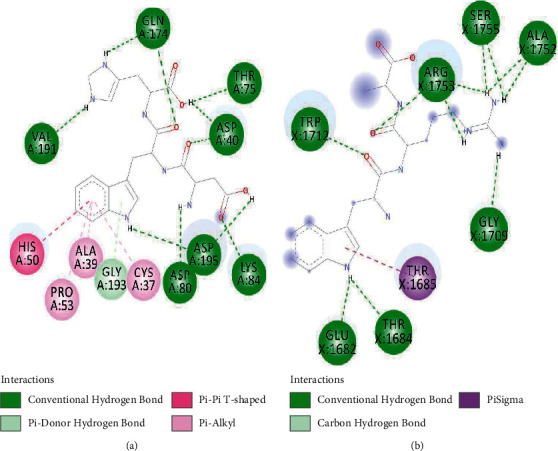
2D diagrams of the closest interactions exhibited by the complexes compound 5-1JIJ (a) and compound 6-1JNX (b), which showed the most significant molecular interactions.

**Figure 6:**
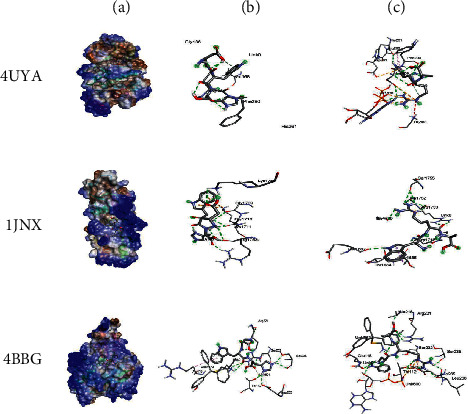
3D illustrations of the selected honey compounds (5 and 6), which possessed the highest binding scores and the targeted receptors (a); the corresponding closest 3D interactions for compounds nos. 5 (b) and 6 (c).

In order to better understand the mechanistic effects underlying the biological effects of the compounds identified in AH, the binding affinities and molecular interactions of several receptors involved in these biological activities were assessed. The selection of the reported positions was based on the best binding score and RMSD equal to zero, as is commonly reported in the existing literature [22–24]. As presented in Table 5, while all the constituent compounds of AH exhibited negative binding energies (ranging from −3.8 to −11 kcal/mol) with the different targeted receptors, the best scores were obtained for the complex compound no. 1 and the JIJ receptor. The number of conventional hydrogen bonds, the number of the closest interacting residues, and the closest distance during the ligand-receptor complex formation are presented in Table 6. It was predicted that compound no. 5 established 12 conventional hydrogen bonds with the TyrRS for *S. aureus* tyro-syl-tRNA synthetase (1JIJ). This was the highest number of H-bonds revealed in this study. Interestingly, 12 closest interacting residues were also observed in the same complex (Figures 4and5), among which Asp195 was the closest (2.037 Å). Moreover, it was predicted that compound no. 6 established 11 conventional H-bonds with 1JNX and evolved 8 closest interacting residues. The distance to Arg1753 was just 2.020 Å, which confirmed that the compound was deeply embedded. Overall, the tripeptides (compound nos. 5 and 6) and aminocyclitol glycoside (compound no. 1) appeared to exhibit better activities toward the different targeted receptors compared to the other classes of compounds. This was particularly true for the tripeptides (Asp-Trp-His and Trp-Arg-Ala) (Figures 4 and 5) (see Figure 6).

## 4. Discussion

The present study reports eight dominant phytochemicals and three small peptide-like proteins identified in AH using the LC-MS technique. Several phytochemicals have been reported previously in AH [25–28]. The techniques such as liquid chromatography together with UV detection and liquid chromatography-electrochemical detection (LC-ECD) are often employed to determine the contents of phenolic acids in honey samples [26, 29–31]. In 2014, Wang and colleagues demonstrated that the AH collected from beekeepers in the region of Shaanxi (China) was rich in chlorogenic acid, p-hydroxybenzoic acid, ellagic acid, gallic acid, syringic acid, rosmarinic acid, and protocatechuic acid [26]. Kaempferol rhamnosides and rhamnosyl glucosides have been reported as the markers for AH [32, 33]. In the present study, a pyridine alkaloid compound named anabasamine was isolated, which has also been reported previously in the chemical composition analysis of *Anabasis aphylla* L. [34, 35]. Anabasamine is reported to possess weak anti-acetylcholinesterase and anti-inflammatory properties [36, 37]. Three small peptide-like proteins (two tripeptides and one dipeptide) were also identified in the present study. Previously, Al Aerjani and colleagues have also used the same technique (LC-MS) and reported identifying short and cyclic peptides in the *A. hamulosa* honey with high medicinal effects, including antioxidant, antimicrobial, and antitumor effects. These peptides were also reported as potential weight loss-inducing peptides [31]. The small peptide-like proteins identified in the honey samples in the present study were two tripeptides (Asp-Trp-His and Trp-Arg-Ala) and a dipeptide (Pro-Arg). Previous studies have demonstrated that peptides containing tyrosine, arginine, tryptophan, methionine, lysine, cysteine, and histidine residues exhibit higher antioxidant activities [38]. Recently, it was demonstrated that tripeptides (Asn-Asn-Asn, His-Phe-Gln, Gln-His-Phe, Thr-Leu-Trp, and Gln-Phe-Tyr) identified in the aqueous and methanolic extracts of *Allium subhirsutum* L. (bulbs) interacted with the structural and nonstructural proteins of SARS-CoV-2 with high binding energy [39].

Several studies have highlighted that honey exhibits antimicrobial potential, particularly against a wide spectrum of pathogenic bacteria, including both Gram-positive and Gram-negative bacteria [40–43]. The emergence of novel drug-resistance bacterial strains has instigated the search for novel phytochemicals with antimicrobial potential. The complexity of the chemical composition of honey and its intrinsic characteristics contribute to the valuable antimicrobial properties that honey exhibits [40]. Several mechanisms and target bacterial sites could be responsible for this antimicrobial property of honey, rendering honey resistance a rare event [41]. The honey that has been most commonly investigated for its antimicrobial potential is Manuka honey. The antimicrobial potential of this honey has mainly been associated with the high value of methylglyoxal (MGO) [42]. A comparative analysis of the antibacterial effects of Manuka honey and AH revealed a valuable result in favor of AH [43]. In addition, AH from various geographical regions has been demonstrated to exhibit interesting antimicrobial potentials in several studies [5]. The studies exploring the proprieties of honey samples from Saudi Arabia have revealed significant variation in their physicochemical characteristics, total phenolic contents, pigments, hydrogen peroxide levels, and dicarbonyl compounds with the botanical origin, climate, and altitudes, particularly for the AH samples from this region [7, 44, 45].

In the present study, the antibacterial activity of AH was observed to vary in a dose-dependent manner, with higher antibacterial activities achieved at 100% AH concentration. At this concentration, the efficiency of AH was higher, with the highest mGIZ value obtained against *S. aureus* (clinical strain), *E. coli* (clinical strain 217), and *S. epidermidis* ATCC 12228 (mGIZ values: 40.67 ± 0.57 mm, 50.33 ± 0.57 mm, and 40.33 ± 0.57 mm, respectively). These results were interesting when compared to the mGIZ values observed for ampicillin, which was used as a reference. A recent study on different kinds of honey from Saudi Arabia revealed that AH exhibited a more potent antibacterial effect against several microbial strains, with higher mGIZ values obtained against Gram-positive bacteria compared to when using other kinds of honey [46]. Moreover, *B. cereus*, *S. aureus*, *E. coli*, and *Salmonella enteritidis* were reported as the most sensitive bacterial species [5, 46]. These findings were consistent with the results of the present study, particularly those obtained for *S. aureus* and *E. coli*. The present study also revealed that the antimicrobial potential of AH was dose-dependent, with the highest activity observed when pure honey was used. In another study, the authors concluded that the antimicrobial activity increased when water-diluted honey (33% w/v) was used rather than nondiluted honey [46]. This could be due to the difference in the honey moisture rate. The most resistant bacteria were *A. baumannii* (clinical strain 146), with the mGIZ value of approximately 6.00 ± 0 mm. A recent study investigating the novel nano-composite hydrogels comprising silver nanoparticles (AgNPs) and AH revealed that these hydrogels exhibited strong bactericidal activity against standard nosocomial strains, while *A. baumannii* exhibited a notable resistance [47]. Therefore, it was inferred that *A. baumannii* could exhibit resistance against AH, although this must be confirmed through further investigation.

Furthermore, the activity of AH against the yeast and molds was almost negligible, except for a moderate activity exhibited against *S. cerevisiae* with an mGIZ value of 20 ± 0 mm, which was higher than the corresponding mGIZ value obtained for amphotericin (7.33 ± 0.57 mm). Mracevic et al. [48] evaluated 20 kinds of honey collected from different regions of Serbia, including AH, against *C. albicans* and reported that none exhibited any potency. Another study evaluated several honey samples, including AH, and reported resistance in 9 fungal strains (*F. oxysporum, A. brasiliensis, A. alternate, D. stemonitis, T. longibrachiatum, T. harzianum, P. canescens, P. cyclopium,* and *C. albicans*) [49]. These findings are consistent with the results obtained in the present study, indicating that most fungal strains could be resistant to honey. The mechanism of this resistance should, however, be deciphered through further investigation.

The microdilution assay performed for twelve bacteria revealed a significant bactericidal activity of AH against *P. aeruginosa* (clinical strain SP-40), *E. coli* ATCC 10536, *E. coli* (clinical strain 217), *S. aureus* (clinical strain), *A. baumannii* (clinical strain 146), and *S. aureus* MR (clinical strain 136) strains, with the obtained MBC/MIC ratio of 2. In the case of the yeast and fungi, fungistatic effects were observed mainly, with the MFC/MIC ratio of ≥4, which could be the reason for the fungal resistance to AH. Previous studies on the antimicrobial effects of the AH obtained from different regions against a wide variety of bacteria, yeast, and mold strains have reported that the corresponding MIC and MBC/MFC values vary with the chemical composition of the honey sample evaluated. Stojkovska and colleagues [47] reported that AH collected from Serbia in 2018 exhibited antimicrobial activity against a huge collection of Gram-positive and Gram-negative bacteria, and also against certain fungal strains. These authors reported MIC values ranging from 25 mg/mL for *B. cereus* ATCC 10876 strain to >100 mg/mL for *E. coli* ATCC 25922, *P. aeruginosa* ATCC 10145, *S. typhimrium* ATCC 14028, *S. epidermidis* ATCC 12228, *K. pneumoniae* ATCC 70063, *B. subtilis* ATCC 6633, *E. faecalis* ATCC 29212, and *Micrococcus lysodeikticus* ATCC 4698. The AH from Serbia also exhibited anti-*C. albicans* activity with an MFC value of >100 mg/mL [49]. Moreover, Yousaf and colleagues [50] reported that the AH collected from Malaysia exhibited activity against *S. aureus*, *E. coli*, *S. typhimirium*, *P. aeruginosa, Listeria monocytogenes*, *Clostridium jejuni* ATCC 29428, and *B. cereus*, with the MIC values ranging from 25% to 50%, while the MBC values ranged from 50% to >50%. A comparison of the results obtained in the present study with the findings of previous studies revealed that AH exhibits an efficient antimicrobial activity against most bacterial strains, particularly the Gram-positive bacteria [51, 52]. The variation in the antimicrobial effect of AH observed in several studies could be attributed mainly to the variations in the composition and constituent phytochemical compounds of AH, which are, in turn, influenced by the botanical origin and the physicochemical properties (osmotic effect, pH, and the presence of undefined molecules with antimicrobial effect) of the honey sample [52–54]. Therefore, AH could be a valuable alternative treatment option for the pathologies associated with drug-resistant bacterial strains. However, the incomplete knowledge regarding the constituent bioactive phytochemicals and their mechanisms of action creates another level of complexity in the use of AH for the treatment of infections. Such reasons have limited the application of honey in conventional medicine in the absence of standardization of antibacterial activity [55, 56].

Since honey is derived from plants, it contains several phytochemicals that confer antioxidant properties to honey. The antioxidant potential of honey is due to the presence of bioactive compounds, such as phenols, flavonoids, and tannins, which are capable of inactivating the free radicals generated during diverse cellular processes. Accumulation of free radicals could have a cytotoxic effect or maybe potentially carcinogenic. In the present study, the free radical-scavenging activity of AH was investigated using three approaches: DPPH radical-scavenging activity, ABTS radical-scavenging activity, and *β*-carotene/linoleic acid method. These three methods revealed IC_50_ values of 0.670 mg/mL, 1.065 mg/mL, and >5 mg/mL, respectively, with the highest scavenging activity observed in the DPPH assay. These results were comparable to those obtained for butylated hydroxytoluene (BHT) and ascorbic acid (AA), which were used as references. Therefore, it was inferred that AH derived from the Hail region exhibited significant antioxidant potency. In addition, the total phenolics content (TPC, 6.546 mg GAE/g), total flavonoid contents (TFC, 0.400 mg QE/g), and tannin contents (5.352 mg TAE/g) obtained were interesting. According to a previous report, the AH from Ordu in Turkey exhibited an IC_50_ value of 24.53 ± 1.26 mg/mL when using the DPPH approach, with the estimated total phenol content of 51.91 ± 1.32 mg/100 g GAE [57]. In another study, the AH from central Serbia exhibited an antioxidant potential with IC_50_ = 8.36 ± 0.42 mg Trolox/kg honey, total phenolics content = 68.48 ± 5.53 mg GAE/kg honey, and flavonoid content = 18.59 ± 1.71 mg QUE/kg honey [49]. The AH from the Hail region used in the present study, comparatively, exhibited a higher antioxidant activity. On the contrary, the total phenolics content (TPC) and total flavonoid content (TFC) observed for Malaysian AH were relatively higher (79.08 mg GAE/1 mg and 20.98 mg CE/1 mg, respectively) compared to the AH used in the present study, indicating that the antioxidant properties of the former would enable inhibiting the growth of breast cancer cells through apoptosis [58]. However, even though the AH from the Hail region contained lower levels of TPC and TFC compared to Malaysian AH, higher anticancer activity was observed for this AH in the MTT assay. The TPC and TFC contents may vary with the geographical and botanical origins of the honey samples. A strong antioxidant capacity of AH may, therefore, not be related to the phenolics, flavonoid, or tannin contents only. The phytochemical profiling in the present study revealed the presence of various chemical compounds, such as polypeptides, in the AH, and the synergies among these compounds could also be involved in the mechanism underlying the antioxidant activity of this honey.

Several *in vitro* and *in vivo* studies have demonstrated the anticancer activity of various kinds of honey against different types of cancers. Raw honey contains high levels of polyphenols and flavonoids, which are considered the main factors contributing to antioxidant and anticancer effects. Studies have also demonstrated the efficiency of honey as a chemo-protectant or an adjuvant in cancer treatment [59]. In addition, certain phytochemicals derived from honey exert a therapeutic effect for the treatment of various types of cancer and are, therefore, to be considered prominent chemo-preventive agents [60]. There could be several mechanisms underlying the anticancer effect of honey, including cell cycle arrest, induction of apoptosis, modulation of the mitochondrial pathway, membrane permeabilization, and anti-inflammatory and immunomodulatory effects [2]. AH is considered a potential therapeutic candidate for both the prevention and treatment of cancer. This potential of AH, however, varies in the degree of effectiveness with the floral source of AH and/or the geographical regions from which the AH samples are derived [61].

In the present study, the anticancer potential of the AH from the Hail region of Saudi Arabia was evaluated based on the MTT assay using 3 cancer cell lines: breast cancer (MCF-7), lung cancer (A549), and colon cancer (HCT-116) cell lines. All three cell lines were treated with different concentrations of AH, and it was observed that the proliferation of cancer cell lines was inhibited in a dose-dependent manner. The IC_50_ values obtained for AH against the breast, colon, and lung cancer cell lines were 5.053 *μ*g/mL, 5.382 *μ*g/mL, and 6.728 *μ*g/mL, respectively. These results demonstrated a cytotoxic effect of the AH from the Hail region against cancer cells. In a previous investigation, doxorubicin was reported to exhibit an IC_50_ value of 1.2 ± 0.036 *μ*g/mL for the HCT-116 cell line and 1.09 ± 0.044 *μ*g/mL for the MCF-7 cell line [7]. Another study investigating the AH from the Asir region (southwest of Saudi Arabia) reported a notable cytotoxic effect against HCT-116, MCF-7, and HepG2 cell lines. The authors also reported that the AH from different altitudes of this region exerted different degrees of *in vitro* effects on the cancer cell lines [7]. The high-altitude AH exhibited higher activity against human cancer cell lines compared to that exhibited by the low-altitude AH, and there was a noticeable difference in the total phenolics and flavonoid contents as well. In the present study, AH exhibited a higher anticancer activity against HCT-116 and MCF-7 cancer cell lines, which could be attributed to the differences in the composition of AH from the Hail region and that from the Asir region.

Previous studies have also highlighted that AH from Malaysia inhibits the growth of breast cancer cell line MCF-7 via apoptosis. The induction of apoptosis occurred after 2 h, while the formation of the apoptotic bodies could be detected within 6 h of the AH treatment [58]. The IC_50_ value of AH from Malaysia was 5.49 *μ*g/mL after 72 h, while that of the AH used in the present study was 5.053 *μ*g/mL after just 24 h of treatment, indicating the higher cytotoxic activity of the latter.

Another study targeting the lung cell cancer line NCI-H460 revealed that AH could inhibit cancer cell proliferation and cause cell cycle arrest at G0/G1 phase. In addition, it was demonstrated that AH might induce the downregulation of bcl-2 and p53 genes [62]. This cytotoxic effect was also observed in the present study against the lung cancer cell line A549.

Recently, studies have revealed that generating silver (Ag) nanoparticles in the presence of AH (Abha, Saudi Arabia) and the plant extract of *Calotropis procera* produces exhibit a synergic effect with prominent anticancer potentials through the inhibition of the liver cancer cell line HepG2 growth with good immunostimulatory effect [63]. Earlier studies have reported that the anticancer potential of honey may vary with the subtype of honey, even when the same honey from different geographical regions is used. This variation is attributed to the variations in the constituents of the honey samples [64]. The anticancer properties of honey have been attributed mainly to the phytochemicals present in the honey, particularly the flavonoids and phenolic acids. These phytochemicals confer cancer prevention and treatment abilities by interfering in several cellular pathways [65]. In the present study, interesting anticancer activities were demonstrated by AH against three cancer cell lines. In addition to attributing this anticancer activity to the quantitative evaluation of flavonoids and phenolic acids, we should also consider other bioactive compounds that might be contributing to the qualitative constitution of honey. In the present study, tripeptides and aminocyclitol glycoside present in the AH from the Hail region were observed to contribute to enhancing the biological potency of honey.

Furthermore, the binding affinities and molecular interactions of the constituents of honey were assessed against several receptors involved in the studied biological activities (Figure S1). One of these receptors is 1JIJ, which is a *S. aureus* tyrosyl-tRNA synthetase and is commonly recognized to provide a structural basis for designing novel antimicrobial agents [66, 67]. Another one is 2 × ct, which is a type IIA topoisomerase that cleaves and then ligates DNA strands to regulate the DNA topology. Type IIA topoisomerases represent a major class of antibacterial and anticancer drug targets [68]. The receptor 2QZW is an aspartic proteinase Sap 1 secreted from *Candida albicans,* which reportedly plays a key role in superficial Candida infections [69]. The human peroxiredoxin 5, 1 hd2, reduces hydrogen-and alkyl hydroperoxides and is implicated in the antioxidant protective mechanisms and cellular signal transduction [70]. The MLK4 kinase domain 4uya regulates the JNK, p38, and ERK kinase signaling pathways. Mutations in MLK4 have been detected in several cancers [71]. The receptor 1JNX is the C-terminal BRCT region of BRCA1 and is essential for DNA repair, tumor suppressor functions, and transcriptional regulation [72]. The receptor 4bbg is the mitotic kinesin Eg5 and is critical for the assembly of the mitotic spindle and a promising chemotherapy target [73]. All the compounds present in honey exhibited negative binding energies (ranging from −3.8 to −11 kcal/mol) with the different targeted receptors. As reported in several studies, the variation in the binding affinity could mainly be attributed to the chemical structure of the compound [22, 74, 75]. Usually, there is a strong relationship between the structure and the activity of a compound, which explains the importance of SAR and QSAR analyses. In the present study, the best binding score was obtained for the 1JIJ-aminocyclitol glycoside complex. The tripeptide Asp-Trp-H is formed 12 conventional H bonds with the TyrRS in the *S. aureus* tyrosyl-tRNA synthetase (1JIJ). The closest molecular interactions included 12 residues in the active site. Aminocyclitol glycoside was deeply embedded in the active site of the different targeted receptors (1.864–2.617 Å) and was at a distance of only 2.037 Å from the Asp195 residue in 1JIJ, for which the highest binding affinity was reported. The closely related ligand-amino acids/protein complexes could explain the biological activity [21–24]. The compound 6 (Asp-Trp-His) was predicted to form 11 conventional H-bonds with 1JNX and evolve 8 closest interacting residues. It was at a distance of only 2.020 Å from Arg1753, which satisfactorily explained the potential biological activity. Furthermore, the molecular interactions of the different complexes usually included certain key residues associated with the pharmacological effects [67, 74, 75]. Overall, the biological effects of the phytochemical compounds in honey appeared to be thermodynamically feasible, particularly those of the tripeptides and aminocyclitol glycoside. These computational results in parallel with the findings of *in vitro* analysis could explain the promising effects of AH and its ethnopharmaceutical use worldwide, particularly in the Middle East region. Owing to the complexity of the composition of AH, there is incomplete knowledge regarding its constituent phytochemicals and their mechanisms of action, which is the main reason for the limited application of honey in conventional medicine in the absence of standardization of biological activities. Therefore, further studies are required to isolate and characterize each compound for a better understanding of the involved mechanism of action.

## 5. Conclusions

The present study reports the potential biological activities of the AH from the Hail region of Saudi Arabia. A broad-range bactericidal effect of AH against a wide spectrum of clinically relevant bacterial strains was observed. On the other hand, only fungistatic activity was observed for the yeast and the molds. A notable cytotoxic effect of AH was observed on the three cancer cell lines evaluated. Among the identified phytochemicals, the tripeptides and aminocyclitol glycoside exhibited considerable biological effects of enhancing the antimicrobial, anticancer, and antioxidant potentials of AH. In addition, huge amounts of polyphenols and flavonoids were detected in the evaluated honey samples. Nonetheless, further studies on the isolation, characterization, and assessment of these biological activities of the compounds in AH in the present study should be conducted to support the above-stated findings. The findings of the present study demonstrate the potential of AH as a source of natural bioactive compounds that could be used for therapeutic purposes.

## Figures and Tables

**Table 1:** Phytochemical compounds identified by the HR-LCMS technique in AH from Hail region.

No	Compound name	Chemical class	RT	MW	Chemical formula	[*m*/*z*]−	[*m*/*z*]+
1	6-(alpha-D-Glucosaminyl)-1D-myo-inositol	Aminocyclitol glycosides	0.929	341.1312	C_12_ H_23_ N O_10_	—	342.1385
2	L-Gulonate	Sugar acid	1.062	196.0579	C_6_ H_12_ O_7_	195.0507	—
3	Pro-Arg	Dipeptide	1.223	271.1647	C_11_ H_21_ N_5_ O_3_	—	294.1539
4	Anabasamine	Alkaloid	1.666	253.1543	C_16_ H_19_ N_3_	—	276.1435
5	Bakankoside	Glycoside	3.038	357.144	C_16_ H_23_ N O_8_	—	380.133
6	Asp-Trp-His	Tripeptide	3.922	456.1732	C_21_ H_24_ N_6_ O_6_	—	457.1808
7	Trp-Arg-Ala	Tripeptide	12.066	431.230	C_20_ H_29_ N_7_ O_4_		432.237
8	Palmitic amide	Fatty acid amide	17.145	255.2559	C_16_ H_33_ N O	—	256.263
9	Stearamide	Fatty acid amide	18.765	283.287	C_18_ H_37_ N O	—	284.2942
10	10,16-Heptadecadien-8-ynoic acid, 7-hydroxy, (E)	Fatty acid	20.188	278.1935	C_17_ H_26_ O_3_	277.1865	—
11	14-Fluoro-myristic acid	Fatty acid	27.191	246.2011	C_14_ H_27_ F O_2_	291.1997	—

*Note.* RT: retention time (mn); MW: molecular weight (g/mol); [*m*/*z*]−: mass-to-charge ratio in negative ionization mode; [*m*/*z*]+: mass-to-charge ratio in positive ionization mode.

**Table 2:** Growth inhibition zone values expressed in mm of *Acacia* honey tested against bacteria, yeast, and molds strains using well-diffusion assay.

Bacteria tested	Dilution tested				Ampicillin (10 mg/ml)
	25%	50%	75%	100%	
	GIZ ± SD^*∗*^	GIZ ± SD	GIZ ± SD	GIZ ± SD	GIZ ± SD

*P. aeruginosa* (clinical strain, SP-40)	9.67 ± 0.58f	11.00 ± 1.00h	12.67 ± 0.58f	14.00 ± 100f	6.00 ± 0.00h
*S. aureus* ATCC 29213	20.67 ± 0.58d	24.33 ± 1.53d	26.00 ± 2.65d	31.33 ± 1.15c	31.00 ± 1.00c
*S. epidermidis* ATCC 12228	23.33 ± 1.15c	27.00 ± 1.00c	30.00 ± 2.00c	39.33 ± 1.15b	27.33 ± 1.15e
*E. coli* ATCC 10536	9.33 ± 1.15f	12.00 ± 1.00h	14.33 ± 0.58f	14.33 ± 1.15f	28.67 ± 1.15d
*K. pneumoniae* (clinical strain, 140)	9.33 ± 0.58f	13.33 ± 1.15g	14.00 ± 1.00f	15.33 ± 1.15e	6.00 ± 0.00h
*E. coli* (clinical strain, 217)	29.00 ± 1.00b	32.67 ± 2.08b	37.33 ± 1.15b	38.33 ± 1.53b	31.67 ± 0.58c
*S. aureus* (clinical strain)	31.33 ± 1.15a	35.00 ± 1.00a	43.00 ± 1.00a	48.33 ± 1.53a	39.33 ± 1.154b
*S. sciuri* (environmental strain)	15.00 ± 1.00e	18.33 ± 1.53f	25.33 ± 1.15d	31.00 ± 1.00c	50.67 ± 1.154a
* S. marcescens* (clinical strain)	7.67 ± 0.58g	9.33 ± 1.15i	14.00 ± 1.00f	13.00 ± 1.00f	6.67 ± 0.58h
*A. baumannii* (clinical strain, 146)	6.00 ± 0.00h	6.00 ± 0.00j	6.00 ± 0.00g	6.00 ± 0.00g	21.00 ± 1.00f
* E. cloacae* (clinical strain, 155)	6.00 ± 0.00h	13.00 ± 1.00g	14.67 ± 1.15f	17.00 ± 1.00d	12.67 ± 0.58g
*S. aureus MR* (clinical strain, 136)	14.00 ± 1.00e	20.67 ± 0.58e	23.00 ± 1.00e	29.67 ± 1.53c	26.00 ± 1.00e

Yeasts and molds tested	Dilution tested				Amphotericin B (10 mg/ml)
	25%	50%	75%	100%	
	GIZ ± SD	GIZ ± SD	GIZ ± SD	GIZ ± SD	GIZ ± SD

*C. albicans* ATCC 20402	6.00 ± 0.00a	6.00 ± 0.00a	6.00 ± 0.00b	6.00 ± 0.00b	14.00 ± 0.00a
*S. cerevisiae* (instant yeast)	6.00 ± 0.00a	6.00 ± 0.00a	14.00 ± 1.00a	18.67 ± 1.15a	7.67 ± 1.54d
*C. guilliermondii* ATCC 6260	6.00 ± 0.00a	6.00 ± 0.00a	6.00 ± 0.00b	6.00 ± 0.00b	13.00 ± 1.00a
*C. tropicalis* ATCC 1362	6.00 ± 0.00a	6.00 ± 0.00a	6.00 ± 0.00b	6.00 ± 0.00b	12.00 ± 1.00b
*C. vaginalis* (clinical strain, 136)	6.00 ± 0.00a	6.00 ± 0.00a	6.00 ± 0.00b	6.00 ± 0.00b	7.33 ± 0.57d
*A. fumigatus* ATCC 204305	6.00 ± 0.00a	6.00 ± 0.00a	6.00 ± 0.00b	6.00 ± 0.00b	11.00 ± 0.00c
*A. niger*	6.00 ± 0.00a	6.00 ± 0.00a	6.00 ± 0.00b	6.00 ± 0.00b	6.00 ± 0.00e

^*∗*^: growth inhibition zone ± standard deviation (expressed in mm); the letters (a–h) indicate a significant difference according to the Duncan test (*p* < 0.05).

**Table 3:** MICs, MBCs/MFCs expressed in mg/ml, and MBCs/MIC, MFC/MIC ratio of *Acacia* honey tested against bacteria, yeast, and molds using microdilution assay.

Test systems	*Acacia* honey	(BHT)	(AA)

Tested microorganisms	*Acacia* honey		MBC/MIC ratio
	MIC	MBC	

*P. aeruginosa* (clinical strain SP-40)	150	300	2
*S. aureus* ATCC 29213	75	600	8
*S. epidermidis* ATCC 12228	75	600	8
*E. coli* ATCC 10536	150	300	2
*K. pneumoniae* (clinical strain 140)	75	300	4
*E. coli* (clinical strain 217)	150	300	2
*S. aureus* (clinical strain)	150	300	2
*S. sciuri* (environmental strain)	75	600	8
*Se. marcescens* (clinical strain)	75	300	4
*A. baumannii* (clinical strain 146)	300	600	2
*Enterobacter cloacae* (clinical strain 155)	150	>600	>4
*S. aureus* MR (clinical strain 136)	300	600	2

Tested microorganisms	*Acacia* honey		MFC/MIC ratio
	MIC	MFC	

*C. albicans* ATCC 20402	150	600	4
*S. cerevisiae* (instant yeast)	150	>600	>4
*C. vaginalis* (clinical strain 136)	300	>600	>2
*C. guilliermondii* ATCC 6260	150	>600	>4
*C. tropicalis* ATCC 1362	150	>600	>4

**Table 4:** Antioxidant activities of *Acacia* honey sample as compared to butylated hydroxytoluene (BHT) and ascorbic acid (AA).

Test systems	*Acacia* honey	(BHT)	(AA)
Total flavonoids content (mg QE/g) extract)	0.400 ± 0.053c	—	—
Total tannins content (mg TAE/g) extract)	5.352 ± 0.964 b	—	—
Total phenols content (mg GAE/g) extract)	6.546 ± 0.876a	—	—
DPPH IC_50_ (mg/ml)	0.670 ± 0.015c	0.023 ± 3 × 10^−4^	0.022 ± 5 × 10^−4^
ABTS-IC_50_ (mg/ml)	1.065 ± 0.116c	0.018 ± 4 × 10^−4^	0.021 ± 0.001
*β*-carotene IC_50_ (mg/ml)	5b	0.042 ± 3.5 × 10^−3^	0.017 ± 0.001

The letters (a–c) indicate a significant difference between the different antioxidant methods according to the Duncan test (*p* < 0.05). Data are presented as mean ± SD.

**Table 5:** Binding affinity of the identified compounds in honey (1–11) with the different targeted receptors (1JIJ, 2XCT, 2QZW, 1HD2, 4UYA, 1JNX, and 4BBG).

No	Compounds	Binding affinity (kcal × mol^−1^)						
		1JIJ	2XCT	2QZW	1HD2	4UYA	1JNX	4BBG
1	6-(alpha-D-Glucosaminyl)-1D-myo-inositol	−11.0	−7.7	−8.0	−7.7	−7.9	−7.1	−7.8
**2**	L-Gulonate	−7.8	−5.6	−6.5	−5.6	−6.3	−5.8	−7.3
**3**	Pro-Arg	−8.1	−6.7	−7.3	−6.0	−6.8	−6.1	−6.4
**4**	Anabasamine	−9.4	−6.6	−7.9	−6.4	−6.9	−6.2	−6.3
**5**	Bakankoside	−9.0	−7.0	−8.7	−6.6	−6.8	−6.5	−7.6
**6**	Asp-Trp-His	−8.9	−6.8	−8.2	−6.4	−7.4	−6.1	−7.7
**7**	Trp-Arg-Ala	−5.7	−4.4	−5.2	−4.0	−4.4	−4.5	−4.1
**8**	Palmitic amide	−6.7	−5.3	−6.0	−5.3	−5.0	−4.4	−4.9
**9**	Stearamide	−6.5	−4.3	−5.1	−4.9	−4.7	−4.5	−6.0
**10**	10,16-Heptadecadien-8-ynoic acid, 7-hydroxy, (E)	−6.8	−5.3	−5.9	−5.1	−5.6	−4.0	−4.4
**11**	14-Fluoro-myristic acid	−5.9	−4.7	−4.8	−4.5	−4.5	−3.8	−4.2

**Table 6:** Conventional hydrogen-bonding, the number of closest interacting residues and distance to closest interacting residue (Å) of the compound with best scores (1, 5, and 6) with the different targeted receptors (1JIJ, 2XCT, 2QZW, 1HD2, 4UYA, 1JNX, and 4BBG).

No.	Chemical structure	Receptor	Conventional H-bonds	No. closest interacting residues	Closest interacting residue	
					Residue	Distance (Å)
1	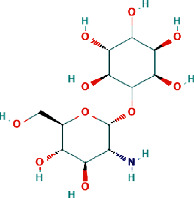	1JIJ	4	6	Thr75	1.864
		2XCT	4	6	Met1113	2.617
		2QZW	5	6	Arg192	2.088
		1HD2	7	4	Arg86	2.139
		4UYA	7	7	Gly346	1.987
		1JNX	6	6	Glu1836	2.028
		4BBG	5	6	Arg221	2.154

5	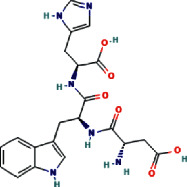	1JIJ	12	12	Asp195	2.037
		2XCT	6	9	Ser1098	2.334
		2QZW	9	7	Thr222	2.179
		1HD2	9	7	Asn76	1.818
		4UYA	4	5	Asp289	1.877
		1JNX	7	5	Gly1710	2.079
		4BBG	8	8	Ser235	2.180

6	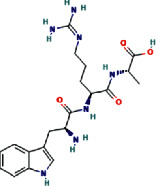	1JIJ	9	11	Asp177	1.891
		2XCT	11	5	Gly1111	1.912
		2QZW	10	12	Thr222	1.840
		1HD2	9	5	Leu140	2.072
		4UYA	7	6	Phe290	2.422
		1JNX	11	8	Arg1753	2.020
		4BBG	10	11	Ala218	1.956

## Data Availability

The data used to support the findings of this study are available from the corresponding author upon request.

## Supplementary Materials

Supplementary Materials Figure S1: The 2D diagrams of the closest interactions exhibited by AH major identified compounds complexed with the different targeted receptors displaying the most significant molecular interactions.
