# Supplementary material for: Chemical Composition and the Anticancer, Antimicrobial, and Antioxidant Properties of Acacia Honey from the Hail Region: The in vitro and in silico Investigation
Source: Evid Based Complement Alternat Med. 2022 Aug 4;2022:1518511. doi: 10.1155/2022/1518511 (PMC9371847; doi:10.1155/2022/1518511)
Supplement: Supplementary Materials — Figure S1: The 2D diagrams of the closest interactions exhibited by AH major identified compounds complexed with the different targeted receptors displaying the most significant molecular interactions. [file 1518511.f1.docx]

Supplementary material


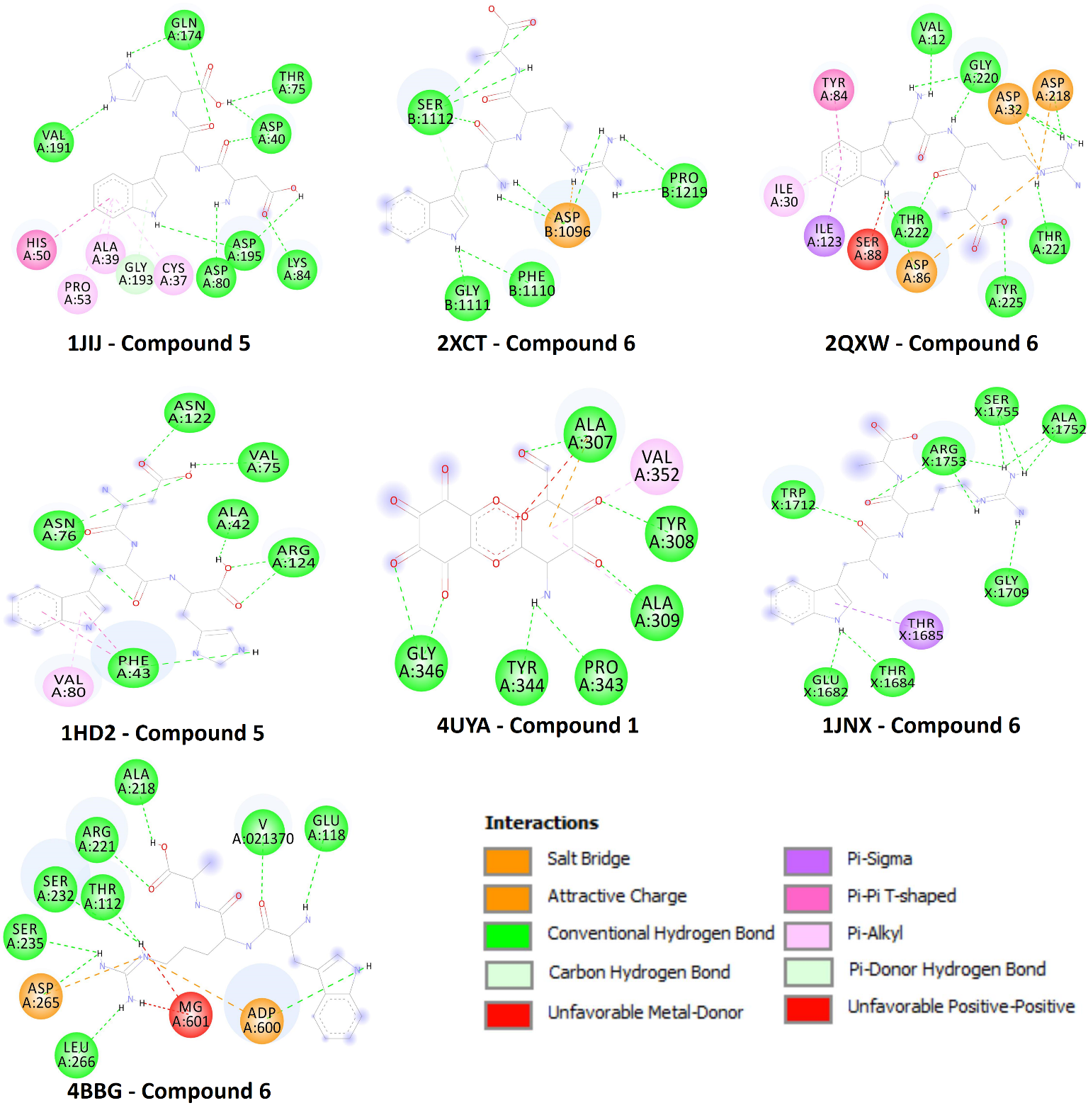


Figure S1: 2D diagrams of the closest interactions exhibited by the honey major identified compounds complexed with the different targeted receptors:1JIJ, 2XCT, 2QXW, 1HD2, 4UYA, 1JNX and 4BBG, which showed the most significant molecular interactions.
